# Obstruction after Sleeve Gastrectomy, Prevalence, and Interventions: a Cohort Study of 9,726 Patients with Data from the Scandinavian Obesity Surgery Registry (SOReg)

**DOI:** 10.1007/s11695-021-05574-9

**Published:** 2021-08-15

**Authors:** Linda Sillén, Ellen Andersson, Torsten Olbers, David Edholm

**Affiliations:** 1 Department of Surgery and Department of Biomedical and Clinical Sciences, Linköping University, Norrköping, Sweden; 2 Department of Surgery and Department of Biomedical and Clinical Sciences, Linköping University, Linköping, Sweden

**Keywords:** Sleeve gastrectomy, Bariatric, Obstruction, Stricture, Stenosis, Complications

## Abstract

**Background:**

Sleeve gastrectomy (SG) is the most common bariatric procedure worldwide. Obstructive symptoms, together with leaks, are among the most serious postoperative complications. This study aimed to investigate the incidence of symptomatic obstruction after SG in Sweden and to explore risk factors, treatment strategies, and outcome.

**Methods:**

A retrospective analysis of prospectively collected data from the Scandinavian Obesity Surgery Registry (SOReg) of patients undergoing SG and developed obstruction symptoms within the first postoperative year was performed. For patients who had undergone any re-intervention, such as endoscopic dilatation or remedial surgery, medical charts were reviewed.

**Results:**

From 2007 to 2018, a total of 9,726 SG were performed, and 59 (0.6%) of them developed postoperative obstruction. Intolerance of solid food was the most common symptom associated with obstruction (80%). Sixty-one percent of the patients had obstruction at the level of incisura angularis. Longer operative time, higher rate of perioperative complications, longer hospital stay, and oversewing the staple line were associated with an increased risk of obstruction. Endoscopic balloon dilatation was performed in 59% of patients (n=35) and successful in 18 patients (51%). Twenty-one patients (36%) underwent surgical conversion to Roux-en-Y gastric bypass (RYGB). After revisional surgery, 11 (52%) reported complete relief of symptoms.

**Conclusions:**

Obstruction was rare (0.6%) and most often located at the incisura angularis. Obstruction was associated with longer operative time, perioperative complications, oversewing of the staple line, and longer hospital stay. Endoscopic dilatation or surgical conversion to RYGB frequently alleviates symptoms, but despite treatment, almost 50% reported residual symptoms.

**Graphical abstract:**

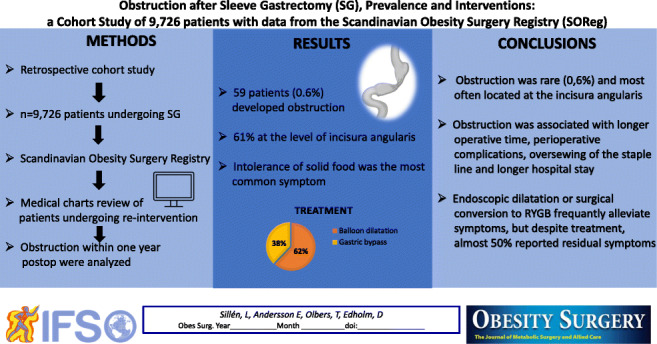

## Introduction

In 2016, more than 1.9 billion adults had overweight and 650 million obesity [1]. Bariatric surgery is the most successful treatment for severe obesity, and it is associated with long-term weight loss and reduction of weight-related comorbidities [2–4]. Several bariatric procedures have been used over the years, and currently, sleeve gastrectomy (SG) is the most popular bariatric procedure [5]. In Sweden, RYGB has been the predominating method of bariatric surgery, but since 2019, SG accounts for more than half of the bariatric procedures [6].

Although SG is considered to be technically easier and with shorter operative time compared to RYGB, SG is not without complications, such as bleeding, staple line leak, fistulas, and strictures [7–9]. Obstruction is, together with leaks, one of the most feared complications post sleeve gastrectomy. Obstruction is often attributed to a stricture or stenosis of the gastric sleeve, or a distorted gastric anatomy resulting in functional obstruction. The two most common obstructive conditions are believed to be caused by different mechanisms: (1) a mechanical narrowing, usually located at the incisura angularis, and (2) axial obstruction due to rotation phenomenon secondary to incongruence between the anterior and posterior gastric wall [8, 10]. Gastric sleeve obstruction has been reported to occur in 0.2–4% of cases and most cases of obstruction present within 6 weeks after surgery [7, 11–15]. Predisposing factors are considered to be smaller bougie diameter, stapling too close to the incisura angularis, postoperative edema, or hematoma [8, 9]. The incisura angularis is the most prevalent location for obstruction [8, 9, 14, 16]. Symptoms of obstruction can be nausea, vomiting, dysphagia, abdominal pain, reflux, food intolerance, and/or rapid weight loss [8, 10, 11, 15, 17].

Treatment options for obstruction after SG are endoscopic balloon dilatation, stent placement, and conversion to for example Roux-en-Y gastric bypass. Endoscopic treatment is reported to be successful in 88–94% of cases [15, 18–20]. When endoscopic methods are unsuccessful, conversion to RYGB should be considered [8, 13, 15, 16, 20].

This study aimed to investigate the incidence of symptomatic obstruction after sleeve gastrectomy in Sweden and, additionally, explore risk factors, treatment strategies, and outcome.

## Materials and Methods

This is a retrospective analysis of prospectively collected data from the Scandinavian Obesity Surgery Registry (SOReg). SOReg is a nationwide Swedish quality registry for bariatric surgery. The registry covers >98% of all bariatric procedures in Sweden and is continuously validated [6].

All patients in the register, who underwent a sleeve gastrectomy between 2007 and 2018, were assessed regarding documented postoperative symptoms related to obstruction (nausea and vomiting, eating problems, abdominal pain, dysphagia, and gastro-esophageal reflux) within the first postoperative year. For patients who had information in SOReg about undergoing any re-intervention, such as endoscopic dilatation or remedial surgery, medical charts were reviewed. The charts were requested from the surgical units through letters, and if no charts were returned, centers were contacted by phone or e-mail. Patients who had the sleeve gastrectomy as a first-stage operation in a two-step bilio-pancreatic diversion with duodenal switch procedure were excluded. After constructing a chart of parameters from SOReg and medical records, the following variables were analyzed:

- Demographic/clinical parameters (age, sex, weight, body mass index (BMI), comorbidities, smoking status)
- Surgical parameters (operative time, surgical access, oversewing of the staple line, bougie size, intraoperative complications)
- Postoperative data (perioperative complications within 30 days; leaks, bleeding, deep vein thrombosis/pulmonary embolism, pneumonia, length of hospital stay, follow-up time, need for nutritional support)
- Obstruction-related data (time to diagnosis, type of obstruction, location of obstruction, diagnostic work-up; upper gastrointestinal contrast study, computed tomography (CT), upper gastrointestinal (GI) endoscopy)
- Treatment-related/outcome data: type of endoscopic or surgical management, time to first dilatation after surgery, the number of balloon dilations, balloon size, stenting, success rate associated with endoscopy, revisional surgery data, improvement of symptoms, weight development, and follow-up data

Demographic and surgical parameters were collected from SOReg and postoperative obstruction data from retrieved medical records. Success after intervention was defined as alleviation of obstruction-related symptoms assessed by medical records. Results are presented as median (range) or mean ± SD (standard deviation), minimum and maximum for continuous variables, and frequencies (percentage) for categorical variables. Continuous variables were analyzed using independent sample *t*-test or a nonparametric test, as appropriate. Categorical variables were compared using Chi square, or Fisher’s exact test. Statistical significance was defined as *p* < 0.05. Statistical analysis was performed using SPSS version 26.0 (IBM SPSS Statistics, Armonk, NY, USA).

## Results

**Fig. 1:**
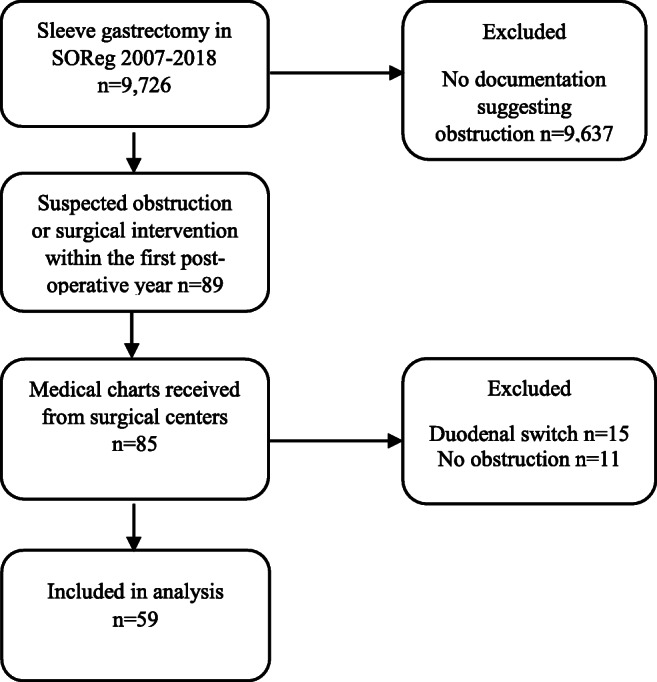
Flowchart visualizing the inclusion and exclusion of study subjects

SOReg included 9,726 registered patients who had undergone SG during the time period of 2007–2018. The register contains 1-year follow-up data of 80% patients. A total of 89 patients were identified with suspected obstruction requiring intervention, and 85 medical charts were received from 24 different Swedish bariatric centers. After review of the 85 medical records, 59 patients with obstruction following SG were identified and were included in the analyses. A flowchart is presented in Fig. 1. Patients having SG as a first-stage operation in a two-step bilio-pancreatic diversion with duodenal switch and patients with lack of data confirming that they in fact had an obstruction were excluded from analysis.

Given that 9,726 SG were performed during the time period, 0.6% (n=59) of them developed postoperative obstruction. Demographics for the entire SG cohort (n=9,726) as well as the study group are given in Table 1. The study group consisted of 50 women (85%) and 9 men with a mean age of 39.8 years (range 18–58). Preoperatively, their mean weight was 108.8 kg (range 81.8–160) and BMI 38.2 kg/m^2^ (range 32.4–47.3). The most frequent preoperative comorbidities were diabetes mellitus 13.5% (n=8) and hypertension 13.5% (n=8). All patients had a laparoscopic procedure with a median operative time of 52 min (range 17–184). Mean hospital stay was 3.0 days (range 1–10). Median follow-up time in the study group was 12 months (range 2–95 months). The mean BMI of the patients with obstruction 1 year after the initial surgery was 26 kg/m^2^ (range 19–38) and the mean postoperative weight was 74.5 kg (range 52–125). Bougie sizes ranged between 30 and 40 French, with a median size of 34.5 French. Staple line oversewing was performed in 39 patients (66.1%) of patients with obstruction. The method of oversewing varied between different hospitals.

**Table 1:** Comparison between the group of patients with obstruction and the entire group operated with sleeve gastrectomy.

	Obstruction group (n= 59)	Total SG group (n = 9,726)	*P* value
Mean age (years)	39.8 ± 10	41.2 ± 11	0.328
Mean weight preop (kg)	108.8 ± 17.1	112.8 ± 21.3	0.152
Mean BMI preop (kg/m^2^)	38.2 ± 3.5	39.6 ± 5.8	0.314
Female	84.7%	80.5%	0.408
History of smoking	12.3%	11.8%	0.248
Diabetes	13.5%	9.1%	0.724
Hypertension	13.5%	20.3%	0.427
Periop complications 30 d	19.1%	4.7%	< 0.001
Laparoscopic access	100%	99.2%	0.930
Bougie size, mean (Fr)	34.3 ± 1.9	34.4 ± 2.1	0.817
Staple line oversewing	66.1%	46.4%	0.042
Operative time, mean (min)	61.1 ± 30.4	47.5 ± 21.3	0.002
Mean hospital stay (days)	3.0 ± 2.1	1.6 ± 2.3	0.003
Mean weight 1 year postop (kg)	74.5 ± 15.1	83.3 ± 17.8	0.001
Mean BMI 1 year postop (kg/m^2^)	26.0 ± 3.5	29.2 ± 5.1	< 0.001

*BMI* body mass index. *Fr* French. *P* value <0.05

Patients with obstruction had significantly longer operative time, higher rate of oversewing of the staple line, higher rate of complications, and longer postoperative hospital stay. Weight and BMI at 1-year postoperative follow-up were lower in patients with obstruction than in patients without obstruction.

**Fig. 2:**
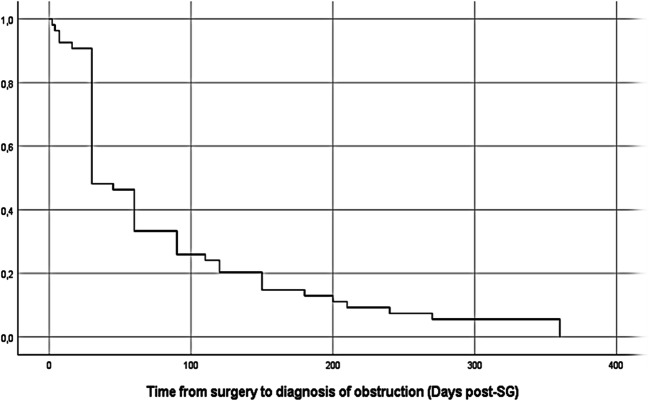
Time from SG to diagnosis of obstruction

The median time from SG to diagnosis of obstruction was 30 days (range 2–360) (Fig. 2). Early obstruction (diagnosed within 6 weeks from SG) represented 56% (n=30) of the cases. Sixty-one percent (n=36) of the patients had obstruction at the level of incisura angularis and 10% (n=6) a more proximal obstruction at the level of hiatus. Two patients were diagnosed with a twist of the gastric tube in the immediate postoperative period. In 25% (n=15) of cases, localization of the sleeve obstruction was not stated in the charts.

**Fig. 3:**
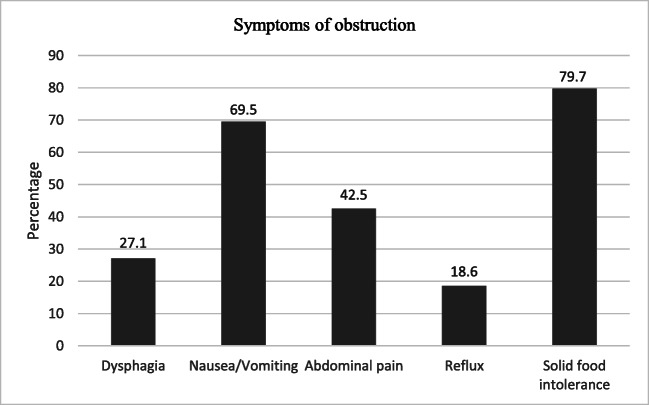
Percentage of patients presenting with different symptoms

The most common symptom was intolerance of solid food 79.7% (n=47), followed by nausea/vomiting 69.5% (n=41). Other symptoms reported by patients with obstruction were dysphagia, reflux, and abdominal pain (Fig. 3).

Intraoperative complications occurred in two patients (3.4%) with obstruction, represented by intra-abdominal bleeding and in one who had an allergic reaction to anesthesia. Nine (19%) of patients had perioperative complications within 30 days, which included hemorrhage (n=2), pneumonia (n=2), wound infection (n=1), leak (n=1), gastric ulcer (n=1), small bowel obstruction (n=1), and intra-abdominal abscess (n=1).

Gastroscopy was the most common diagnostic procedure, performed in 52 (88.1%) patients with obstruction. Other diagnostic methods used were upper gastrointestinal study with oral contrast 69.5% (n=42) and abdominal CT-scan 50.8% (n=30).

The majority of patients with symptoms of obstruction n=45 (80%) required one or more interventions. Endoscopic balloon dilatation was performed in 35 patients (59.3%), most commonly (n=15) with a 20-mm balloon, and for the rest (n=10) with a ≥30-mm balloon. In 10 patients, the size of the balloon was not reported. There was no difference in success rate related to balloon size. The average number of endoscopic dilatations was 2.9 (range 1–13) (Table 2).

**Table 2:** Data on interventional procedures performed to alleviate obstruction

	Mean ± SD	Range
Time to first dilatation after SG (days)	190 ± 260	15–1250
Number of balloon dilatations	2.9 ± 2.8	1–13
Balloon size (mm)	24.2 ± 6.5	15–40
Time from SG to revision surgery (days)	604 ± 507	110–1800

*SD* standard deviation, *SG* sleeve gastrectomy

Overall, 56% (n=25) reported complete resolution of their symptoms following either endoscopic treatment or revisional surgery. Residual symptoms (vomiting, dysphagia, malnutrition, and chronic abdominal pain) were reported by 20 patients (44%). Nine of these patients had endoscopic balloon dilatation, three had revisional surgery to RYGB, and eight of them were treated with a combination of these. In patients with residual symptoms after surgery, 7 (35%) had chronic pain preoperatively and 5 (25%) had undergone multiple abdominal surgeries prior to SG. Out of the patients who had endoscopic balloon dilatation (n=35), 18 (51%) reported resolution of symptoms, and 17 (49%) had residual symptoms after treatment. Twenty-one patients (35.6%) underwent surgical conversion to RYGB. After revision surgery to RYGB, 11 (52%) reported complete relief of symptoms, whereas 10 (48%) reported having residual symptoms such as nausea, vomiting, chronic abdominal pain, and malnutrition. In those who underwent revisional surgery and experienced residual symptoms, 70% had undergone multiple dilatations and 50% multiple surgical procedures. In five patients (11%), perioperative complications due to endoscopic treatment or revisional surgery were reported. Reported complications included pain, wound infections, perforation of the sleeve, incisional hernias, and hemorrhages. There was no mortality.

## Discussion

In this SOReg-based cohort of SG, we found that 59 out of 9,726 developed an obstruction requiring intervention, resulting in an incidence of 0.6%. These data are in line with previous research estimating obstruction incidence to 0.2–4% [7, 11–15].

Several other studies have shown that incisura angularis is the most frequent location of obstruction [8, 9, 11], as was the case in our study. This location seems to be more susceptible to narrowing, possibly due to its angular shape where the linear staple line can result in a locus minoris resistentiae for kinking as well as risk for true stenosis if stapling too close to incisura angularis.

The patient group presenting with obstruction had a high rate of complications within 30 days (19%) and longer mean hospital stay after SG (3.0 days) in comparison to the mean hospital stay after primary bariatric surgery (1–2 days) [6]. A plausible explanation is that the patients had longer hospital stay because of obstruction symptoms appearing early in the postoperative period. Median time to definite diagnosis was 30 days. We found an association between longer operative time and risk of obstruction. Additionally, the high rate of overall perioperative complications indicates that complications may predispose to development of sleeve obstruction. However, if certain types of complications predispose for obstruction is beyond the scope of this study. In a registry study from Germany of 11,800 patients, the total postoperative complication rate was 5%. The study found that a longer operation time was associated with an increased frequency of complications, such as staple line leaks [21]. Similar results were seen in a recently published large registry study on 389,839 patients from Canada concerning major postoperative complications and prolonged operation time [22].

Our results indicate that oversewing of the staple line may constitute a risk factor for obstruction after SG since 66% of patients with obstruction had a staple line oversewing. Similar results were seen in Burgos study; oversewing was associated with increased stenosis risk [23]. Rebibo et al [15] also reported that all patients with stenosis had a partially or fully sutured staple line. Thus, it is possible that an inadvertently placed and deep suture can cause a stricture of the gastric tube. In the study by Shelton et al [22], no association between oversewing of the staple line and development of sleeve stenosis was detected.

The review of the medical records showed that most surgical centers diagnose and treat obstructions in a similar fashion. Swedish surgeons seem to favor the approach of observation followed by less invasive means as endoscopic balloon dilatation as a first-hand treatment. Only later, if necessary, conversion to RYGB is used. Gastroscopy and upper gastrointestinal series are the dominating diagnostic methods. This is in line with recommendations from the international Sleeve Gastrectomy Expert panel in its consensus paper on approach to sleeve obstruction/stenosis. The expert panel recommends observation, followed by endoscopic dilatation attempts and reoperation if >6 weeks with failed endoscopic treatment [8]. With new diagnostic tools such as 3D CT reconstruction and expanding clinical experience, an update of the expert panel consensus on the management of sleeve obstruction may be needed.

Despite treatment, 20 patients (44%) in our study reported residual symptoms (vomiting, dysphagia, malnutrition, and chronic abdominal pain) following interventions. Out of these, 10 patients had been converted to RYGB. Medical charts revealed that 35% had chronic pain preoperatively and 50% had undergone multiple abdominal surgical procedures prior to conversion. These figures indicate that RYGB was performed as a last resort, which may explain the low success rate.

Out of all the patients treated with endoscopic balloon dilatation (n=35), about half of them reported resolution of symptoms and the other half had persistent symptoms. Shnell et al [24], who also reported the use of a 20-mm balloon, had similar results (44% resolution). Other studies have reported higher success rates of 80% [17] and 87% [15]. Interestingly, Ogra and Kini [11] reported poor response, 11% to controlled radial expansion (CRF) balloon, but after switching to 30-mm achalasia balloons, the success rate increased to 71%. Donatelli et al [25] and Rebibo et al [15] also showed higher success rates using the 30-mm balloon. In Sweden, the standard praxis seems to be to use a smaller balloon size, and in most cases, only one or two dilatations were performed. No correlation between balloon size and success rate was seen in this study.

This study has several limitations. The most important is that the register-based design may lead to an underestimation of the true incidence due to non-recorded interventions, or patients lost to follow-up. The follow-up rate beyond 1 year is 80% and this could have been higher. There is an uncertainty that we may have missed some patients who may have had a later obstruction. However, clinically relevant obstruction after sleeve gastrectomy often requires endoscopic or surgical intervention and the incidence of obstruction found is similar to obstruction rates in previous studies. The register-based study design is, however, also a strength due to a relatively large study group from an entire country’s bariatric production over a substantial time period and thereby represents real-world data. A strength of this study is the 1-year follow-up period for the registry cohort. Though about half of the cases with obstruction present within the first 6 weeks postoperatively, still 44% present later during the first postoperative year. A limitation is the evaluation of symptoms after interventions through information retrieved from medical records. This methodology may underestimate, as well as overestimate, residual symptoms.

## Conclusion

Obstruction after SG was rare (0.6%) in our nationwide study of 9726 patients. Incisura angularis was the most frequent location of obstruction and the majority of patients were diagnosed within the first months after SG. Obstruction was associated with a longer operative time, higher rate of perioperative complications, and a longer hospital stay. Oversewing of the staple line may increase the risk of obstruction in SG.

Endoscopic dilatation or surgical conversion to RYGB is frequently effective to alleviate symptoms, but despite treatment, almost 50% in our study reported residual symptoms.
